# Enhancing Healthcare Services for Vulnerable Aging Populations: A Comparative Analysis of Puerto Rico and International Case Studies

**DOI:** 10.3390/healthcare14070829

**Published:** 2026-03-24

**Authors:** Varun Nannuri, Sara Belligoni, Darya Sulkouskaya, Rutwa Shah, Om Pathak, Fernando I. Rivera

**Affiliations:** 1 Burnett School of Biomedical Sciences, College of Medicine, University of Central Florida, Orlando, FL 32827, USA; 2 Puerto Rico Research Hub, University of Central Florida, Orlando, FL 32816, USA; 3 John Jay College of Criminal Justice, The City University of New York, New York, NY 10019, USA

**Keywords:** healthcare systems, aging population, physician migration, Puerto Rico, Cuba, Japan, the Philippines, South Korea

## Abstract

This study examines healthcare system strains in rapidly aging societies through a comparative analysis of Puerto Rico, Cuba, Japan, the Philippines, and South Korea. While existing research documents global aging and physician migration trends, few studies explore how these challenges manifest in conjunction with each other. Puerto Rico presents a critical case, with 24% of its population aged 65+, severe physician migration, and systemic underfunding under U.S. Medicaid structures. Using a structured comparative case methodology, we analyze policy responses across four nations with divergent approaches: Cuba, Japan, the Philippines, and South Korea. Data from government reports, academic literature, and World Health Organization (WHO) datasets show that (1) proactive medical education investments outperform reactive measures, (2) dedicated long-term care financing is essential but structurally unavailable in Puerto Rico, and (3) territorial status in the case of Puerto Rico, constrains policy innovation. Conventional aging frameworks are challenged by revealing how high-income territories can exhibit low systemic adaptability. Proposed are targeted reforms for Puerto Rico, including Medicaid restructuring and workforce incentives, with broader implications for aging societies under constrained sovereignty. This study fills a critical space in understanding how geopolitical contexts shape healthcare system vulnerabilities.

## 1. Introduction

Currently, Puerto Rico is a commonwealth of the United States as it holds a territory status. Its healthcare system models that of the continental United States, including both public and private sectors [1]. However, in recent decades, the healthcare system has undergone major changes and challenges that have put strain on the population of the island. The major avenues through which these effects are seen include decreased availability of care for the aging population, regionalization of healthcare services, and physician migration creating a physician shortage on the island [1]. Understanding how other countries address similar challenges could offer valuable insight, highlighting both successful strategies and mistakes that might be relevant for Puerto Rico’s context.

Puerto Rico’s status as a United States territory introduces structural features that distinguish its healthcare system from those of sovereign nations. As a U.S. territory, Puerto Rico operates under federally determined Medicare and Medicaid frameworks, including capped Medicaid funding that differs from the open-ended financing structures available to U.S. states [2,3]. Additionally, because residents of Puerto Rico are U.S. citizens, migration to the mainland U.S. does not require immigration processing or visa authorization [4]. Consequently, physician and youth migration from Puerto Rico occurs without visa restrictions, national credential revalidation, or bilateral labor agreements that typically regulate international migration systems [5]. For this reason, this study does not treat Puerto Rico as directly equivalent to sovereign states. Instead, the analysis compares structural outcomes such as aging-related strain, workforce depletion, and financing limitations while recognizing that the mechanisms producing these challenges differ according to geopolitical status.

To further understand the origins and drivers of these challenges and to identify potential policy responses, the study initially evaluated twelve countries as potential comparators to Puerto Rico. Countries were first screened based on whether they had implemented policies addressing either rapid population aging, defined in this study as the increasing ratio of adults aged 65 years and older and the resulting strain on healthcare service demand, or significant outward migration of the youth and healthcare workforce. In this analysis, youth and healthcare workforce migration refers primarily to the movement of working-age adults under the age of 24, particularly physicians or other healthcare professionals to locations offering greater economic opportunity, professional advancement, or resource availability.

**Figure 1:**
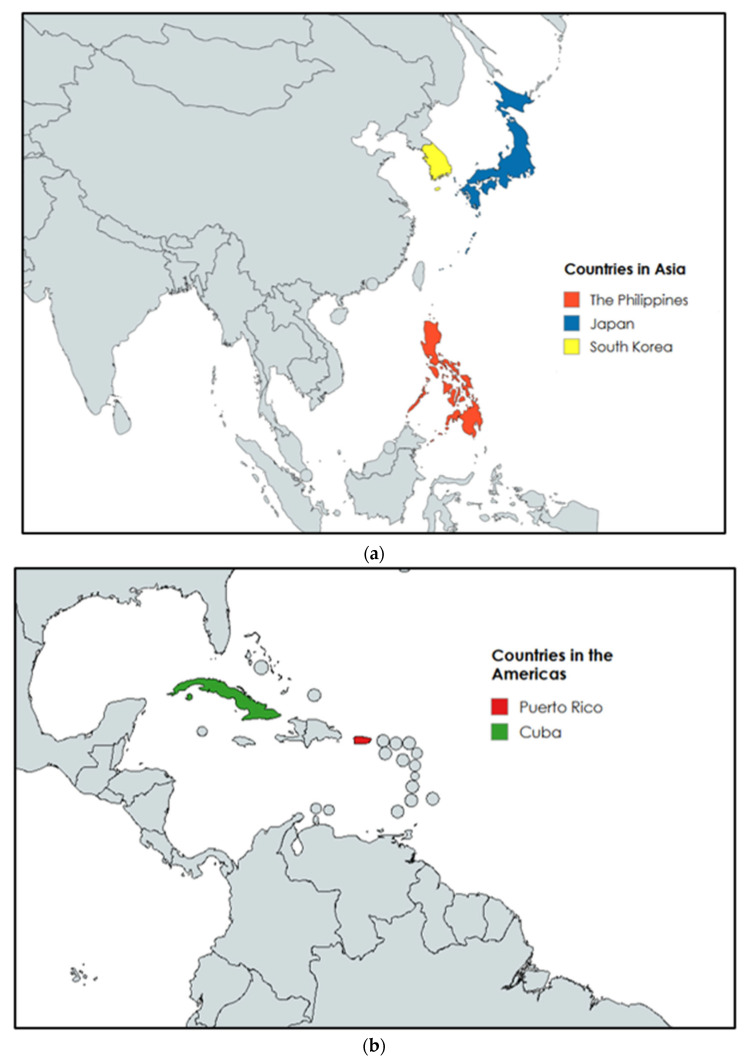
(**a**) Case study selection: Location of the countries selected for comparison with the case of Puerto Rico (Japan, the Philippines, and South Korea). Source: Authors’ elaboration via MapChart.net). (**b**) Case study selection: Location of the countries selected for comparison with the case of Puerto Rico (Cuba and Puerto Rico). Source: Authors’ elaboration via MapChart.net).

The second criteria assessed the relative comparability of healthcare infrastructure and demographic characteristics across candidate countries. Applying these criteria narrowed the pool to four countries that demonstrated both relevant policy responses and sufficient structural comparability. The comparative analysis therefore focuses on how selected countries have attempted to mitigate two intersecting pressures: (1) healthcare system strain associated with aging populations (65+), and (2) healthcare workforce shortages resulting from outward migration of youth (<24) and medical professionals. Particular attention is given to policy interventions such as workforce retention programs, medical education expansion, healthcare financing reforms, and long-term care infrastructure development, as well as the observable outcomes associated with these strategies. The final comparator cases selected for analysis were Cuba, Japan, the Philippines, and South Korea (Figure 1a,b).

Cuba was selected for its history in addressing physician shortages and universal healthcare system with a strong emphasis on preventative care and public health [6,7,8,9]. Japan was chosen due to its similarity to Puerto Rico in terms of an aging population, while also having a well-developed universal healthcare model and long life expectancy [10,11,12,13]. The Philippines was analyzed because of its similar challenges with regional disparities and mixture of public and private healthcare infrastructure [14,15,16]. Lastly, South Korea was chosen due to the multitude of measures taken to address their healthcare problems in regionalization of available medical care and low birth rates [17,18,19,20].

Table 1 provides a general overview of the territorial and demographic traits of each country. All four of them were analyzed regarding their past and present challenges in the healthcare sector, the approaches they have taken to combat these issues, and their insights for Puerto Rican healthcare.

## 2. Literature Review

### 2.1. The Global Aging Population Trend

Globally, populations are aging due to increased life expectancy and decreasing birth rates. In 2020, less than 30% of countries were considered aged societies (with 14% of the population above age 65), and only one country, Japan, will be considered an ultra-aged society (where 21% of the population is above age 65). By 2050, it is projected that these numbers will shift so that 58% of countries qualify as an aged society and 15% will be in an ultra-aged society [21]. As of 2022, the countries and regions with the highest percentages of their population above 65 years old were Monaco, Japan, Saint Helena, Italy, Finland, and Puerto Rico [22]. However, in projections for 2100 based on current trends, the top countries and regions are likely to be Albania, Puerto Rico, American Samoa, South Korea, and Jamaica [22].

This upwards trend in age across the globe can be partially tied to decreasing birth and mortality rates, as public health, biotechnology and medicine continue to improve. The development and accessibility of birth control methods, in addition to measures put in place to decrease infant mortality, have contributed to declining birth rates as well [23]. From 1950 to 2050, the global total fertility rate is projected to drop from 5 children born per woman on average to 2.25. Increases in lifespan and longevity have also contributed to the increase in the average age worldwide. Medical advances and decreases in unhealthy practices, such as tobacco usage, have allowed people to live longer on average, seen in the rise in life expectancy from 47 years in 1950 to 70 years in 2010 [23].

### 2.2. Healthcare Strains Due to Aging Populations

As individuals age, the prevalence of chronic conditions such as diabetes, osteoarthritis, depression, and dementia increases, requiring long-term care and ongoing monitoring, which raises the demand for healthcare services [24,25]. Adults over 65 use medical services at a rate 20% higher than young adults and are hospitalized three times more frequently [26]. When systems cannot meet this rising demand, they become strained. Additionally, the need for long-term care facilities grows, as they are primarily utilized by individuals over the age of 60 [27]. Aging populations are a major driver of rising healthcare expenditures, particularly as long-term care costs begin to surpass in-patient, out-patient, and ambulatory care after age 60 [27,28]. Alongside rising costs, aging populations contribute to labor shortages across sectors, including healthcare. For example, in Japan limited physician availability and a shortage of end-of-life care facilities contribute to unmet healthcare needs [29]. Similarly, Puerto Rico experiences a physician shortage, exacerbated by wage disparities between the island and the mainland United States [30]. Many physicians migrate in search of better resources, wages, and working conditions—a trend seen in Puerto Rico and other countries across the globe. In Puerto Rico’s case, physician migration occurs within a shared U.S. federal labor market and does not require immigration processing [31,32]. This differs structurally from migration patterns observed in countries such as the Philippines or South Korea, where outward mobility involves formal cross-border regulatory processes, bilateral labor agreements, and professional credential and licensure recognition requirements [33,34]. The distinction is analytically important, as Puerto Rico’s territorial status lowers migration barriers and may accelerate workforce depletion relative to sovereign states [31].

### 2.3. Global Physician Migration Trends

Physician migration has profound impacts on the healthcare systems in places across the world, whether from an oversupply of staff to a lack of healthcare providers, which is the more common alternative. In recent years, member countries of the Organization for Economic Cooperation and Development (OECD), a group of 38 countries with developed and emerging economies, have seen increases in physician and nurse migration into their healthcare systems, with healthcare professionals frequently originating from lower- and middle-income countries such as the Philippines and India and migrating toward higher-income countries including the United States, Canada, the United Kingdom, and Australia [5,30]. As a result, low- and middle-income countries often suffer from this outflux of their physicians [30]. Medical personnel have been found to migrate often in search of better career and job opportunities, and compensation, while also moving to avoid safety issues in their home countries including political corruption, rising unemployment, an increase in xenophobia attacks, and instances of murders that target migrants, as seen in a systematic review that saw these trends present across the world, most prevalently in South Africa, India, and the Philippines [33]. These physician shortages in turn impact the quality of care available in these countries, patient wait times, and hospitalization rates, further straining the healthcare system in each country experiencing a shortage [35,36].

To combat the outmigration of healthcare personnel, various countries have implemented approaches to address this and other challenges present in their healthcare systems. Countries including South Korea, Australia, and South Africa, among others, have implemented financial incentives such as loan repayment programs to incentivize providers and nurses to stay [37,38]. More tailored programs have also been developed by countries such as India and the Philippines to expand healthcare resources available in their rural regions [39,40,41]. Programs such as these can serve as models for the future development of initiatives in regions and countries undergoing similar challenges, such as Puerto Rico.

Although aging populations and healthcare workforce migration have been widely studied independently, limited comparative research exists that examines the intersection of demographic aging, outward health workforce mobility, and constrained financing structures, particularly within territorially governed healthcare systems. This study addresses that gap by examining four core variables: healthcare model structure, aging population policies, workforce migration dynamics, and economic sustainability of healthcare systems.

## 3. Methodology

The study employed a comparative case study analysis to gather relevant information from academic papers, news sources, and publicly available data about the healthcare system in Puerto Rico and other cases throughout the world. The authors utilized a structured, targeted scoping approach. Searches were performed in PubMed, Scopus, and Google Scholar using predefined search strings. In addition to the exact strings provided below, modified versions were also applied, including the substitution of synonyms, minor phrasing alterations, and changes in word order to ensure comprehensive retrieval of relevant literature. Search strings were composed of the following terms: “aging population,” “healthcare workforce migration,” “physician migration,” “nursing shortage,” “brain drains,” “healthcare financing,” “healthcare system,” and various country names. Further gray literature was retrieved from official sources including the World Health Organization, the World Bank, OECD, national Ministries and Departments of Health, and United States federal agencies relevant to Puerto Rico’s healthcare system and policy context.

The primary objective of this comparative analysis is to establish a comprehensive background on key areas of interest: the global trend of aging populations, the subsequent healthcare strains experienced in different countries, and patterns of physician migration. These areas were critical for understanding how demographic and workforce challenges interacted to shape healthcare systems worldwide.

To facilitate this analysis, the authors organized each country case study around four standardized areas of comparison:

- Healthcare Model
- Aging Population Policies
- Workforce Migration
- Economic Sustainability of the Healthcare System.

In addition to these four areas of comparison, an initial comparative screening matrix was constructed to assess 12 candidate countries. The matrix included the following standardized indicators: type of healthcare system, programs for the underserved, main crises present, fertility and mortality rates, proportion and migration patterns of youth, healthcare workforce migration dynamics, reasons for migration, regional disparities in care, and workforce attraction policies. Each country was assessed across these variables to determine their relevance and potential contribution to the study’s objectives.

Each selected case study was examined to determine which of these factors apply, allowing the authors to assess the extent to which each case was similar or dissimilar to Puerto Rico. By structuring the data in this manner, the authors could effectively identify patterns and differences that emerged across cases.

The magnitude and presence of these factors within each country were analyzed as the authors assessed the degree of aging-related demographic shifts, physician shortages or migration challenges, and the overall accessibility and distribution of healthcare services. Additional attention was given to regional disparities, as differences in healthcare access within countries provided insight into how geographic and socio-economic variations affected healthcare delivery. By systematically comparing these factors across different countries, the authors could identify cases that share key characteristics with Puerto Rico and highlight specific areas where Puerto Rico’s healthcare system faced unique challenges. This structured approach ensured a comprehensive understanding of various healthcare systems, allowing the authors to draw meaningful conclusions regarding Puerto Rico’s position within global healthcare trends. Information extracted from peer-reviewed studies, policy documents, datasets, and news sources were incorporated into the aforementioned matrix. A deductive analysis was then conducted, where synthesis was performed through comparative cross-case charting and repetitive evaluation among the authors to ensure consistency in interpretation.

The authors initially selected 12 countries for the initial comparative study—Canada, South Korea, Australia, South Africa, India, the Philippines, Germany, Italy, Japan, Cuba, Uruguay, and Colombia—based on the similarities or differences their healthcare systems share with Puerto Rico regarding physician shortages, medical service availability, and other issues. Following this initial selection, they evaluated two different rationales to determine which countries they would focus on. The first rationale was to consider countries that have attempted to solve the problems of either an aging population or of mass youth/workforce migration. The second rationale was to assess the healthcare infrastructure and demographic comparability of countries. These rationales allowed structured reduction of the 12 countries by identifying cases that demonstrated (1) simultaneous aging-related strain and healthcare workforce migration dynamics, (2) policy responses within at least two of the four standardized areas of comparison, and (3) sufficient publicly available and peer-reviewed data. Countries were excluded when they either provided redundant policy models already represented by another case, lacked the combined interaction of demographic pressures and workforce migration central to this analysis, or did not offer enough data for structured comparison.

**Figure 2:**
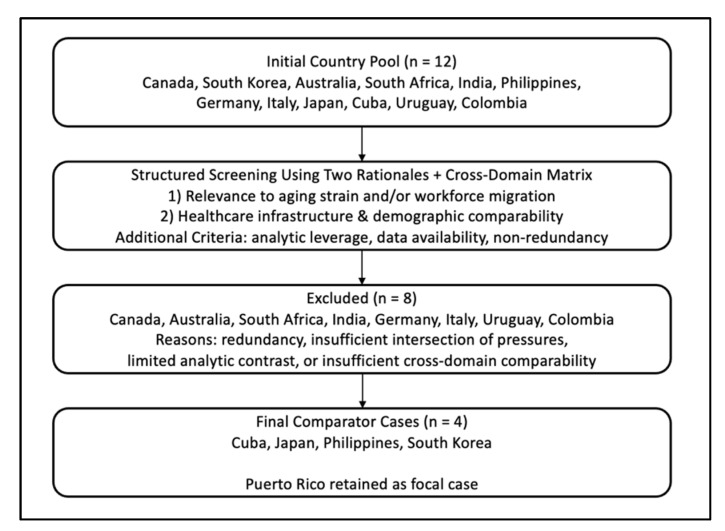
Case selection flow diagram for comparative analysis that demonstrates the structured reduction in the initial 12-country screening pool using predefined rationales. Source: Authors’ elaboration via Microsoft PowerPoint.

Ultimately, the countries that were selected for further analysis were Cuba, Japan, the Philippines, and South Korea. To enhance transparency and reproducibility of the case selection process, Figure 2 illustrates the structured reduction from the initial 12-country screening pool to the final four comparator cases. These countries were chosen because they face comparable issues such as aging populations; physician shortages; “brain drains,” which is when a country faces an export of skilled workers [42]; and regional healthcare disparities between rural and urban areas, making them ideal for comparison. For example, although Italy exhibits an advanced aging population similar to that of Japan, it was excluded because it did not present the same intersection of aging populations and outward healthcare workforce migration emphasized in the study’s comparative framework, nor did it introduce additional policy contrast beyond Japan within the four analytic areas.

Through this process, the authors aim to identify patterns and approaches that could be applied to healthcare systems. By studying the successes and challenges faced by these countries, they sought to build a more comprehensive understanding of the strains on healthcare systems worldwide, which could potentially inform solutions and drive meaningful change for both Puerto Rico and the world’s future healthcare landscape.

## 4. The Case of Puerto Rico

### 4.1. Healthcare Model

Puerto Rico’s healthcare system models that of the continental United States, including both public and private sectors [1]. However, its structure is weakened by dependence on Medicaid block grants. Puerto Rico’s territorial status means Medicaid funding is capped, unlike in U.S. states where it is open-ended, resulting in fewer benefits, reduced drug coverage, and long wait times [3]. With nearly 40% of the population enrolled in Medicaid, this limitation places severe strain on providers and patients alike [43].

### 4.2. Aging Population Policies

The percentage of Puerto Ricans aged 65 or older is one of the highest in the world. Since 2010, this percentage has nearly doubled from 13% to 24% [44,45]. This percentage would place Puerto Rico at third highest in the world [46]. This demographic shift is driven by youth outmigration and a declining fertility rate, presenting a calamitous scenario for the island’s healthcare systems [45].

Recent estimates place the fertility rate at 0.9 births per woman—half the rate it was in the early 2000s [47]. This rate is significantly beneath the threshold of 2.1, which is necessary for full population replacement [48]. Coupled with migratory factors, a declining birth rate also increases the dependency ratio of the island.

Additionally, life expectancy in Puerto Rico was 82 years in 2024 [49]. While this reflects positive health outcomes, it is coupled with high morbidity, disability, and dependency on care systems, further accentuating the island’s aging profile [4].

### 4.3. Workforce Migration

Youth emigration from Puerto Rico is primarily driven by financial incentives and improved job security. With little barrier to entry to the United States, those of working age are incentivized to earn more on the mainland [31]. Moreover, the median age of migrants in 2022 was 30 years old [50]. In 2023, the unemployment rate of Puerto Rico was almost double that of the United States [46].

With high migration rates of Puerto Rican youth to the mainland U.S., the workforce is shrinking, leaving a shortage of physicians, healthcare workers, and caregivers for the island’s elderly population. Since 2014, between 365 and 500 physicians have left the island each year, exacerbating an already critical shortage of medical personnel [51]. This physician emigration is driven by a combination of economic instability, lack of resources, and rising costs of living, all of which make it difficult for doctors to sustain their practices and personal lives. Additionally, systemic issues within the healthcare system—such as low physician reimbursement rates under Medicaid, institutional corruption, and the increasing control of U.S.-based insurance companies—create an unsustainable working environment, forcing many to seek better opportunities on the mainland [32].

Healthcare providers in Puerto Rico earn, on average, less than half of what their counterparts make in the mainland U.S., while Medicaid reimbursement rates remain significantly lower than those in the states [29]. This pay disparity, combined with a lack of financial incentives and resources, continues to drive the exodus of healthcare professionals and weakens Puerto Rico’s medical infrastructure.

### 4.4. Economic Sustainability of Healthcare System

The economic strain on Puerto Rico’s healthcare system is compounded by political and financial challenges. Puerto Rico’s ongoing financial crisis and massive public debt restrict healthcare funding, making it difficult to invest in medical infrastructure, technology, and workforce retention [29]. The island’s vulnerability to natural disasters further threatens sustainability. Hurricanes Irma and Maria in 2017 caused $160 billion in damage [52], while recovery delays left many patients without life-saving care, contributing to thousands of excess deaths [53]. These crises exposed deep infrastructural fragilities, including power grid failures that left hospitals unable to function [54].

The Puerto Rico Oversight, Management, and Economic Stability Act (PROMESA), enacted in 2016, also reshaped financial management, cutting public services to address debt [55]. These fiscal constraints, coupled with disaster-related collapses, underscore the difficulty of sustaining long-term healthcare delivery under the current territorial and economic framework.

## 5. Case Study Comparison: Cuba

Cuba’s healthcare system is known for its universal coverage and strong focus on preventive, community-based care [8]. Since 1959, Cuba has created a universal healthcare system that offers free medical services to all citizens, despite facing financial obstacles on a federal level, including the United States embargo on trade of food and medical supplies [1,8]. The government has also prioritized funding towards research and biotechnology, investing $1 billion from 1990–1996 in this sector and prompting the creation of the ’The Western Havana BioCluster’ [56]. This overall approach has led to increased life expectancy and reduced infant mortality. However, the system now faces challenges from an aging population, workforce migration and a severe economic crisis, which impact healthcare delivery and population health.

### 5.1. Healthcare Model

The National Healthcare System was established in Cuba to provide universal care for its population. It has achieved several successes since its establishment, including a high physician density and the creation of global medical missions. The physician density in Cuba has reached 9.429 physicians per 1000 people in 2021, according to the World Bank, one of the highest physician-to-population ratios globally [57].

Additionally, Cuba provides international medical relief to developing countries, dispatching over 35,000 medical personnel worldwide, particularly in Haiti and Venezuela [1,58]. However, these medical missions have also led to domestic shortages, especially in rural or specialized areas, occasionally delaying care and reducing service quality [6].

### 5.2. Aging Population Policies

Cuba is experiencing a significant demographic transition with a declining fertility rate paired with an increasing life expectancy. The fertility rate was 4.01 in the span of 1950–1955, hit a low of 1.45 in 2010–2015, and is projected to rise to 1.66 in 2045–2050 [59]. Although projected to increase in the coming decades, the overall trend shows a large decrease in the birth rate.

Meanwhile, life expectancy at birth jumped from 59.40 during 1950–1955 to a projected 84.31 for 2045–2050 [59]. As a result, the percentage of the population aged over 60 years has grown from 7% to a projected 36.3% in 2050, compared to a projected 23% for Latin America overall and 31% among developed countries.

The aging population has led to an increased prevalence of chronic diseases such as diabetes, hypertension, and cardiovascular conditions [1], necessitating long-term management and specialized geriatric care. This places additional strain on medical facilities and resources, which are already limited due to economic challenges.

### 5.3. Workforce Migration

Cuba has historically experienced significant physician emigration. Following the 1959 revolution, approximately 1402 physicians left for the United States, creating a notable gap in care [7]. In response, the Cuban government prioritized medical education, leading to a substantial increase in the number of trained physicians. Yet, international medical missions have at times reduced the supply of providers at home. For instance, when more than 8000 Cuban physicians were withdrawn from Brazil in 2018 after disputes over contractual conditions, the sudden shift disrupted both Cuba and Brazil’s systems [58].

### 5.4. Economic Sustainability of Healthcare System

To manage long-term demographic and workforce pressures, Cuba has invested in aging-related policies and programs. The Programa de Atención al Anciano (Program to Care for the Elderly) was established to improve health outcomes by implementing continuous follow-ups on elderly members via community health centers (policlinicas) [60]. In this model, family physicians first evaluate elderly patients and then refer them to geriatric providers as needed. Similarly, the Family Physician and Nurse Program—established in the 1980s—focuses on preventive care, with teams responsible for 600–800 community members (up to 1500 in some areas), emphasizing early detection of age-related conditions [1].

The Rural Medical Service (RMS), launched in the 1960s and expanded in the 1970s, distributed newly graduated physician volunteers to underserved areas, increasing provider availability in rural communities [1]. The Ministry of Public Health also expanded medical and nursing programs in provincial areas during the 1970s, offered free tuition, and prioritized academic merit in admissions, expanding opportunities for disadvantaged students [1].

Together, these initiatives demonstrate Cuba’s commitment to sustaining its system despite economic sanctions and limited resources. Yet, persistent financial hardship and outmigration continue to challenge the long-term sustainability of healthcare delivery.

Table 2 presents a summary of the case study comparison between Puerto Rico and Cuba, following the main themes/factors discussed in this study.

## 6. Case Study Comparison: Japan

Similar to Puerto Rico, Japan faces significant challenges due to an aging population. While healthcare workforce migration is relatively low, the high prevalence of chronic disease among the elderly continues to strain healthcare resources. A declining birth rate further contributes to this strain, as healthcare workers retire later to meet growing demand. This case study examines how Japan’s aging population impacts its healthcare system and explores government efforts to expand elder care and address the nation’s declining birth rate.

### 6.1. Healthcare Model

Japan has a universal healthcare system combining public insurance with a mix of public and private providers. Coverage is nearly comprehensive, but demographic pressures have led to heavy utilization of long-term care services and greater dependence on geriatric infrastructure [9].

### 6.2. Aging Population Policies

Japan’s population is the most aged in the world, with 27.7% aged 65 or older, projected to reach 38.4% by 2038 [9]. One factor contributing to an aging population is life expectancy, with Japan having the highest in the world: 81 years for men and 87 for women in 2016 [11]. Although these figures reflect strong health outcomes, without a stable fertility rate, they place immense burden on healthcare infrastructure.

The fertility rate is among the lowest globally, at 1.33 [61]. With 2.1 births per woman required for replacement, Japan’s population has steadily declined since the 1970s [10]. Younger generations increasingly cite economic hardship, work pressures, and cost of living as reasons for not having children [19].

A Tokyo-based study indicated that over 90% of adults in Japan aged 75+ suffer from at least one chronic disease, and 80% live with two or more [62]. These health burdens require significant long-term and geriatric care expansion.

Policies to address these challenges began with the “Gold Plan” (1980s), a 6 trillion yen initiative to expand long-term care facilities. Though expanded in 1994, demand soon outpaced capacity [10]. Similarly, the 1982 Health and Medical Services Act encouraged healthy aging and promoted nursing homes, but unmet demand produced widespread dissatisfaction [10,11,12].

Fertility-focused initiatives included mandated parental leave (introduced in 1992 and later expanded) and financial stipends for children up to age 9. However, inconsistent government follow-through limited effectiveness [63].

### 6.3. Workforce Migration

Japan has relatively low emigration of healthcare professionals but suffers severe internal labor strain. By the end of 2025, the country is projected to need an additional 2 million nurses and caregivers [64]. To meet this demand, Japan has relied on foreign workers, especially from Southeast Asia.

Due to workforce shortages, the current healthcare workforce is broadly overworked and delays retirement. This has produced some of the highest rates of burnout and depression among healthcare professionals globally [65]. Rural areas face extended wait times, and many residents increasingly turn to private providers for quicker access [65].

### 6.4. Economic Sustainability of Healthcare System

Japan’s response to demographic pressures reflects both ambition and inconsistency. While initiatives such as the Gold Plan and Health and Medical Services Act expanded eldercare, early underestimation of demand undermined sustainability [10,11]. Fertility-support programs also suffered from uneven implementation and insufficient outcomes [63].

The long-term sustainability of Japan’s healthcare system depends on balancing a shrinking labor pool, rising eldercare demand, and escalating fiscal burdens. Despite substantial government investment, structural demographic decline continues to threaten future viability.

Table 3 presents a summary of the case study comparison between Puerto Rico and Japan, following the main themes/factors discussed in this study.

## 7. Case Study Comparison: The Philippines

The Philippines, like Puerto Rico, faces major healthcare challenges due to labor migration, economic instability, and unequal access to care. High rates of healthcare worker migration, an overburdened system, and limited long-term care for an aging population strain the country’s resources. This case study examines the Philippine healthcare system through labor migration, rural access, and reforms under the Universal Health Care (UHC) Act, comparing these trends with Puerto Rico to highlight shared challenges and differing approaches.

### 7.1. Healthcare Model

The Philippines implemented the Universal Health Care (UHC) Act in 2019, aiming to provide all citizens with equitable access to healthcare services. Key features and challenges include financial constraints and healthcare infrastructure disparities. Despite the expansion of healthcare under the UHC Act, finances remain a critical issue, with highly fragmented pieces across national vs. local governments and the public vs. private sector [14]. Inequities across urban areas and rural and underserved regions persist, due to differences in accessibility to healthcare and allocation of resources [14].

PhilHealth was designated as the national strategic purchaser of health services under the UHC Act, tasked with improving efficiency, equity, and financial protection. While PhilHealth introduced reforms to expand benefit packages and simplify eligibility, implementation has been hampered by payment delays, weak provider engagement, and underinvestment in digital infrastructure [14].

The UHC Act indicated a shift from curative medicine toward health promotion and prevention. The Philippine College of Lifestyle Medicine has advocated for incorporating prevention-focused strategies such as culinary education, agricultural integration, and physician training in telemedicine as part of a stronger primary care model [15].

### 7.2. Aging Population Policies

The Philippines does not yet face the same demographic pressure as Puerto Rico. With just 5.26% of its population aged 65 or older in 2023, it is not expected to become an aging society until 2030 [44,66]. In contrast, Puerto Rico already has 24.0% of its population aged 65+.

Currently, the Philippines relies heavily on families to care for elderly members. However, as employment rates rise and family caregiving capacity decreases, the need for eldercare services is projected to grow. Calls for innovation include proposals to integrate elderly assistance for household tasks, medical needs, and social support into mobile application platforms [67]. These approaches could reduce caregiver burden and promote independence but require investment in infrastructure and digital literacy. 

### 7.3. Workforce Migration

The Philippines is one of the world’s leading exporters of healthcare workers, particularly nurses, to countries such as the United States, United Kingdom, and Saudi Arabia [41]. This “brain drain” undermines the availability of medical professionals domestically, leaving hospitals and clinics understaffed [68]. As of 2020, the Philippines had only 19.7 healthcare workers (physicians, nurses, midwives, dentists) per 10,000 population—far below the WHO benchmark of 44.5 [69].

To mitigate shortages, the government has introduced Bilateral Labor Agreements (BLAs) with countries like Canada and South Korea. These aim to regulate migration ethically but often benefit receiving countries more than the Philippines, since enforceable reinvestment provisions remain weak [34]. Deployment caps were also introduced during the COVID-19 pandemic, limiting health worker emigration to 5000 in 2021 and 7500 in 2022 [70].

While remittances from overseas Filipino workers strengthen the national economy, the loss of skilled professionals continues to erode healthcare delivery. Government attempts at offering financial incentives for retention or repatriation have been limited in impact, and nurses still migrate to destinations outside BLAs, particularly the U.S. and Saudi Arabia [41].

### 7.4. Economic Sustainability of Healthcare System

Sustaining healthcare delivery under these conditions requires addressing both financial fragmentation and rural access disparities. The Philippine National Rural Physician Deployment Program attempted to place physicians in underserved areas, but retention remained low: from 1993–2011, only 18% of physicians stayed in their assigned municipalities [40]. Incentives such as community respect, competitive pay, and career opportunities helped retention, while poor infrastructure and weak government support drove attrition [40].

The Special Health Fund was designed to improve financing mechanisms by supporting population health services, infrastructure, and healthcare worker incentives [71]. Recent rural pilot sites also demonstrated that targeted interventions—including provider network expansion, subsidized transportation, technical training, community engagement, and electronic health record implementation—significantly improved outpatient consultations and reduced out-of-pocket spending [72].

Together, the UHC Act, rural access initiatives, and workforce migration policies represent an ongoing but incomplete effort to achieve sustainable healthcare delivery in the Philippines. Financial fragmentation, migration outflows, and limited infrastructure continue to threaten long-term viability.

Table 4 presents a summary of the case study comparison between Puerto Rico and The Philippines, following the main themes/factors discussed in this study.

## 8. Case Study Comparison: South Korea

South Korea faces a major demographic crisis marked by an aging population and declining birth rates. As a super-aged society, 20% of its population is over 65, a figure expected to reach roughly 40% by 2050—placing significant strain on the economy and healthcare system [73]. Labor shortages, especially in caregiving and healthcare, have emerged due to workforce migration, prompting government action [74]. This case study examines how South Korea’s healthcare policies respond to these challenges, focusing on the National Health Insurance (NHI) system, long-term care, technology, workforce strategies, and legislation.

### 8.1. Healthcare Model

South Korea’s National Health Insurance (NHI) system, established in 1989, provides universal healthcare coverage but is financially strained due to rising costs of elderly care [75]. Contributions from the shrinking working-age population are insufficient to cover expenses, leading to frequent premium increases and reliance on government subsidies [76]. These temporary measures raise questions about long-term solvency [77].

Recognizing the need for aging support, South Korea also launched the Long-Term Care Insurance (LTCI) program in 2008. The LTCI expanded services for the elderly, but shortages of professional care workers and heavy reliance on informal caregivers limit its effectiveness [78]. Rising demand has already outpaced contributions, leading to financial instability [17]. Although roughly 90.9% of respondents report satisfaction with LTCI [79], sustainability remains uncertain as population aging accelerates.

South Korea has also integrated technological innovations, particularly during COVID-19. A machine-learning triage system used patient-generated health data to guide treatment urgency [80]. “CareCall,” a call-based dialog agent, facilitated monitoring of patients remotely and reduced unnecessary hospitalizations [81]. These initiatives optimized delivery during acute crises but do not resolve structural issues of labor shortages, rural disparities, and digital literacy barriers.

### 8.2. Aging Population Policies

South Korea’s demographic crisis is driven by increased life expectancy, a record-low fertility rate (0.78 in 2022), and rapid urbanization [82]. These factors elevate healthcare costs, increase the dependency ratio, and shrink the tax base that sustains social services.

These shifts mirror broader East Asian trends but are especially acute in South Korea, where delayed marriage, smaller families, and gendered social norms accelerate population decline. The scale and speed of change raise urgent questions about how policies will adapt to these cultural and demographic pressures.

### 8.3. Workforce Migration

South Korea has sought to address labor shortages through temporary migration policies. The Employment Permit System (EPS) recruits low-skilled workers from 16 countries to serve in healthcare and caregiving roles, aiming to alleviate shortages and reduce recruitment corruption [83].

While EPS provides short-term relief, it perpetuates instability. Migrant workers often receive lower wages, fewer protections, and limited integration opportunities [84,85,86]. They face barriers to housing, social benefits, and residency [74]. Reports highlight longer working hours, lower pay, restricted union rights, and greater exposure to abuse compared to local workers [87].

This dependence on migrant labor reflects a global trend: high-income nations increasingly rely on foreign caregivers while failing to ensure equitable protections. The result is a segmented labor market that undermines sustainability of care delivery.

### 8.4. Economic Sustainability of Healthcare System

South Korea’s health financing model faces long-term sustainability challenges. The NHI and LTCI, while foundational, are under financial strain from high utilization and insufficient contributions. Subsidies have temporarily bridged gaps, but structural reforms to broaden revenue sources are still needed [76].

Reliance on informal caregiving and temporary migrant workers creates additional fragility, as both are unstable supports for a rapidly aging population. Without systemic reforms to stabilize workforce supply and adapt financing structures, the sustainability of South Korea’s healthcare system remains at risk.

Table 5 presents a summary of the case study comparison between Puerto Rico and South Korea, following the main themes/factors discussed in this study.

## 9. Main Challenges and How Other Countries Are Tackling Them

Puerto Rico’s healthcare system is currently navigating a confluence of crises: an aging population, persistent healthcare worker and youth migration, and a bifurcated healthcare financing structure. These interconnected challenges have exposed systemic weaknesses, worsened by economic instability and disasters, and require urgent, multidimensional responses.

### 9.1. Healthcare Workforce Retention and Development

The Philippines faces similar issues with the global export of nurses and doctors. To manage this “brain drain,” it has implemented bilateral labor agreements (BLAs) with ethical recruitment policies, and deployment caps [88]. As it remains federal territory of the United States, Puerto Rico is unable to manage its own migration policy without some level of federal oversight [89]. However, it can explore retention strategies, such as location-based pay incentives and specialized rural healthcare fellowships, modeled after rural physician programs in the Philippines and Cuba. Similar strategies were seen to be effective in Cuba after the revolution in 1959 to combat the migration of skilled professionals out of the country. These initiatives included expanding medical education opportunities and decreasing the financial burdens of medical education for students [1].

Promoting island-based medical practice can also help Puerto Rico with worker retention. Acts 20 and 22, which promote corporate investment and immigration to Puerto Rico respectively, can potentially be re-used to provide benefit to long-term residents of the island [90]. However, expansion of these policies may exacerbate Puerto Rico’s existing wealth divide, as Act 60 beneficiaries enjoy preferential tax treatment while local residents face comparatively higher effective tax burdens [91].

South Korea and Japan, on the other hand, have spent money and resources attracting healthcare workers to the country. However, this model is not sustainable due to temporary visas and a lack of worker protection and integration. With high turnover, South Korea has an unstable workforce [92]. To mitigate this issue, investing resources into incentivizing and providing migrant workers with fair rights and wages would attract foreign healthcare workers in a more stable manner. Japan has extended retirement ages and encouraged older adults to remain in the workforce to sustain its healthcare workforce among rising demand [12]. While Puerto Rico faces more migration-driven workforce shortages, similar retention strategies—paired with burnout prevention policies involving flexible work hours and mental health support—may enhance provider longevity and reduce system strain.

### 9.2. Aging Populations

Cuba faced a healthcare worker shortage in the 1960s due to the outflux of young people following political turmoil and currently faces decreasing fertility rates coupled with high life expectancies [1]. However, the response seen in improving medical education system and embedding providers in communities has aided this transition. On the other hand, the Philippines, though younger demographically, does not have as robust of long-term care models in place for their elderly populations [93]. Japan stands on the opposite of this trend with an increasing older population with a concerning imbalance of age within their population [94]. To combat this and the burden it holds for healthcare, they have implemented financial incentives for boosting the fertility rate and put forth significant funding to expand long-term care in healthcare, as seen in the Health and Medical Services Act [95]. Additionally, through investment in geriatric facilities and home-based chronic care program in areas of dense populations, Japan seeks to treat its aging population [96]. Lastly, South Korea’s aging crisis is driven by low fertility, longer lifespans, and rural depopulation, causing an increase in the demand for long-term care amid a shrinking workforce [97].

Based on the challenges faced by other countries and their responses due to shifting demographics, Puerto Rico, with a high elderly population, should invest in community-based care, a promotion of medical education and an integration of foreign workers within the healthcare system of the island. To help stimulate these solutions, Puerto Rico can focus on monetary and economic incentives to help attract the personnel needed for relief of the healthcare burden. Such incentives include offering caregiver support stipends, tax credits, and caregiver training programs to expand home-based elder care and fill gaps in medical care and attention.

### 9.3. Public vs. Private Healthcare Financing

South Korea’s National Health Insurance (NHI) and Long-Term Care Insurance (LTCI) programs illustrate the impact of universal coverage frameworks [16]. Despite their own fiscal challenges, they provide a model for centralized, equitable healthcare access. The Philippines’ Universal Health Care (UHC) Act also offers lessons in how a national system and strategic investments can reduce disparities [12].

While Puerto Rico may not have full autonomy to reform Medicaid, it can incentivize more doctors, specifically those that have private practices, to accept Medicaid—especially for aging and low-income populations. This can lead to better coordination of care across the island. Additionally, reformation of the Medicaid-capped Block Grant would make significant impacts on services available to the residents. Although these solutions would make healthcare services more accessible economically, the physical restriction of a lack of healthcare infrastructure would still render physical issues, such as appointment waiting time, unresolved.

### 9.4. Rural Access and Community Based Care

Through the Philippine National Rural Physician Deployment Program and Special Health Fund, the Philippines has piloted rural healthcare models that offer targeted support for remote areas [88]. Additionally, Cuba’s Rural Medical Service has supplied a more reliable healthcare workforce for remote areas [1]. Puerto Rico could implement rural incentive programs mimicking the Philippines and Cuba that would address rural provider shortages by providing incentives for healthcare workers and subsidizing population and individual health services.

Preventative healthcare strategies in the Philippines include telemedicine, preventative lifestyle medicine, and community health promotion [68]. Puerto Rico could adopt similar programs, particularly regarding nutrition and chronic disease management, to reduce hospital overload and better serve its elderly population. Telehealth with providers throughout the continental United States could address accessibility and systemic load issues. Adopting South Korea’s advancements in AI-assisted healthcare and telemedicine could serve as a blueprint for Puerto Rico to improve service delivery, which could help overcome workforce limitations [98].

Cuba’s Family Physician and Nurse Program embeds providers and nurses directly into communities, to expand primary care for its populations [1]. A similar model applied to Puerto Rico to define geographic zones could aid in providing care for rural and elderly-dense regions. In these areas, promoting community-based elder care can draw inspiration from Japan’s Health and Medical Services Act. Puerto Rico could leverage its cultural emphasis on family by offering caregiver support stipends, tax credits, and caregiver training programs to support home-based elder care.

## 10. Discussion

Puerto Rico’s healthcare system is experiencing a healthcare crisis because of demographic decline, economic hardship, and structural deficiencies [99]. Nearly one-quarter of the island’s population is now aged 65 or older—placing it among the highest globally—while a record-low fertility rate of 0.9 and emigration of younger individuals and healthcare professionals have intensified systemic pressure [44,47]. These intersecting demographic and workforce changes have hampered the island’s capacity to maintain equitable service, particularly in rural and underserved communities. The comparative cases examined in this study, Cuba, Japan, the Philippines, and South Korea, demonstrate that aging-population related strain and workforce depletions are not unique to Puerto Rico but instead reflect broader global healthcare challenges. However, the policy responses adopted by these countries illustrates distinct approaches to managing vulnerability among countries with aging populations.

Cuba’s model of community-based, preventive care demonstrates how localized service delivery can ease hospital overcrowding and extend care to underserved populations. The country’s sustained investment in medical education and rural service commitments has helped stabilize its healthcare workforce, offering a contrast to Puerto Rico’s ongoing loss of 365 to 500 doctors per year [51]. Although Cuba operates within a different political system and context, its emphasis on prevention and geographically distributed care provides insight into strengthening primary care capacity.

Japan’s action clearly highlights the importance of proactive reform. With similar demographic pressures to Puerto Rico, Japan launched long-term care reforms through its “Gold Plan,” which expanded chronic care capacity, caregiver support programs, and introduced foreign labor integration strategies [9]. While not without implementation challenges, these efforts illustrate the benefits of sustained policy attention to elder care and workforce resilience.

The Philippines has responded to labor losses by establishing bilateral labor agreements, rural fellowship initiatives, and a Universal Health Care framework emphasizing prevention and telemedicine [14]. Although Puerto Rico does not regulate cross-border migration in the same manner due to its status as a territory of the U.S., it could adopt similar tools such as rural service incentives and expanded telehealth infrastructure to retain health workers and expand rural access. With a fertility rate even lower than Puerto Rico’s, South Korea has implemented long-term care insurance, embraced telemedicine, and relied on migrant labor to fill workforce gaps. Their combination of these methods illustrates how aging societies can diversify service delivery models to ensure better quality care for their citizens. However, concerns regarding equitable treatment of migrant labor underscore the importance of ethical workforce policies and continued improvement of these strategies.

Taken together, these cases suggest that healthcare sustainability in aging societies depends on three key foci: (1) strengthening community-based and preventative care infrastructure, (2) stabilizing and incentivizing the healthcare workforce, and (3) ensuring financing mechanisms that can adapt to demographic shifts. Puerto Rico’s territorial governance context shapes how these foci can be implemented, particular regarding Medicaid funding structures. However, comparative analysis indicates that proactive workforce retention strategies, telehealth expansion, and strengthened primary care networks remain viable options for enhancing healthcare services for countries facing aging and vulnerable populations.

## 11. Limitations

This study has a few notable limitations. First, the analysis relies on secondary data drawn from peer-reviewed literature, government reports, and publicly available datasets, which vary in reporting standards and coverage across countries. Although a structured evidence set was used to enhance cross-case consistency, full source equivalence cannot be assumed. In addition, reliance on secondary sources may introduce reporting biases that differ across national contexts, which could influence the availability or emphasis of certain policy outcomes described in the analysis.

Second, the study employs a deductive content analysis rather than quantitative modeling. Therefore, the findings identify structural patterns and policy approaches but do not establish causal relationships or measure effect sizes. As such, the results should be interpreted as analytically informative rather than statistically generalizable, with potential transferability primarily to healthcare systems facing similar demographic and workforce pressures rather than to all national healthcare contexts.

Third, Puerto Rico’s status as a United States territory introduces governance and fiscal constraints distinct from sovereign nations used in the comparison. While this distinction is explicitly acknowledged and even treated as analytically relevant, direct comparability remains inherently limited.

Fourth, the comparative case selection process may introduce a degree of researcher selection bias. Although the study employed predefined criteria, such as the presence of aging-related demographic pressures, healthcare workforce migration dynamics, and relevant policy responses, to guide case selection, the final inclusion of comparator countries ultimately required interpretive judgment by the research team. To reduce this bias, an initial screening process of twelve candidate countries was used before narrowing the analysis to four comparator cases that demonstrated the strongest alignment with the study’s analytical framework. Finally, the Philippines presents a unique case of complexity because it is a nation composed of more than 7000 islands, resulting in substantial regional variation in healthcare infrastructure, workforce distribution, and service accessibility [100]. The present study therefore focuses primarily on national-level policies and structural healthcare trends reported in the literature rather than specific regional health systems within the country. Consequently, this case should be specifically interpreted as a representation of national policy responses rather than a comprehensive analysis of localized healthcare conditions across all regions of the country.

## 12. Conclusions

Puerto Rico’s healthcare challenges reflect a broader global pattern of aging populations interacting with workforce migration and healthcare financing strain. However, the island’s demographic trajectory and constrained fiscal structure have intensified these pressures, placing older adults and rural communities at heightened vulnerability.

The comparative findings underscore that enhancing healthcare services for aging populations requires integrated, multifaceted strategies that combine community-based care, workforce stabilization, and adaptable financing systems. Lessons from Cuba, Japan, the Philippines, and South Korea demonstrate that early investment in long-term care infrastructure, retention incentives for providers, telemedicine expansion, and equitable funding reforms can mitigate demographic stress.

For Puerto Rico, strengthening healthcare resilience will require both internal policy innovation and structural reconsideration of healthcare financing arrangements. By adapting proven international approaches to its unique territorial context, Puerto Rico can move toward a more sustainable, equitable, and responsive healthcare system for its aging and vulnerable populations.

Future research should evaluate the implementation outcomes of specific retention and financing reforms and explore workforce projection modeling to anticipate long-term demographic shifts.

## Figures and Tables

**Table 1:** Territorial and demographic overview of the countries selected for comparison with the case of Puerto Rico (Cuba, Japan, the Philippines, and South Korea). Source: Authors’ elaboration via online available data and governments’ websites.

Country	Puerto Rico	Cuba	Japan	Philippines	South Korea
**Continent**	Americas	Americas	Asia	Asia	Asia
**Area**	13,792 km^2^	110,860 km^2^	377,975 km^2^	300,000 km^2^	100,363 km^2^
**Population**	3,285,874	11,089,511	123,440,000	109,035,343	52,081,799
**Population 65+** **(percentage of total)**	24%	16%	30%	5%	18%
**GDP per capita**	$37,930	$18,329	$32,859	$4,439	$62,960
Gini *	58 (high)	38 (medium)	33.4 (medium)	40.2 (medium)	33.3 (medium)
HDI **	0.845 (high)	0.764 (high)	0.920 (high)	0.710 (high)	0.929 (high)
**Healthcare System**	Private and Public	Universal Health Care	Private and Public	Private and Public (UHC)	Universal Health Care

* The GINI coefficient is a statistical measure to represent income inequalities; it ranges from 0 to 1, with 0 indicating equality and 1 inequality (therefore, the higher the value, the more inequality is present); for simplicity, it is often represented via values from 0 to 100, like in our table above. ** The HDI, or Human Development Index, is a statistical composite index that expresses human development based on data related to education, income per capita, life expectancy. It ranges between 0 and 1, with 0 indicating no human development and 1 maximum human development.

**Table 2:** Comparison of healthcare models and affiliated demographic trends between Cuba and Puerto Rico. Source: Authors’ elaboration via online available data and governments’ websites.

Factor	Cuba	Puerto Rico
**Healthcare Model**	Free universal public healthcare; significant investment in biotechnology and research	US-based Medicaid and Medicare; limited access for undocumented residents; constrained technological capacity due to budget restrictions
**Aging Population Policies**	Community-based primary care approach; Program to Care for the Elderly	Limited eldercare services with reliance on family caregiving
**Workforce Migration**	Large post-revolution physician exodus: government expanded medical education to offset shortages	Ongoing mass emigration leading to severe healthcare worker shortages
**Economic Sustainability of Healthcare System**	Economic hardship persists, but state allocates substantial resources to sustain healthcare delivery	Financial instability from economic decline; heavy reliance on U.S. federal aid

**Table 3:** Comparison of healthcare models and affiliated demographic trends between Japan and Puerto Rico. Source: Authors’ elaboration via online available data and governments’ websites.

Factor	Japan	Puerto Rico
**Healthcare Model**	High healthcare demand for long-term and geriatric care facilities; universal system under strain from aging population	High demand across public and private facilities; public services less reliable than private care
**Aging Population Policies**	Rapidly aging population with low fertility rate; government countermeasures to expand long-term care facilities and promote healthy aging	Rapidly aging population with migration partially offsetting low fertility; reliance on family-based care for the elderly
**Workforce Migration**	Limited outward workforce migration, but shortages persist due to insufficient replacement and high burnout rates	High levels of healthcare worker migration to the United States, worsening domestic shortages
**Economic Sustainability of Healthcare System**	Heavy fiscal strain from rising long-term care costs; repeated government investment but underestimation of demand threatens sustainability	Fiscal instability from economic decline and capped Medicaid funding; heavy dependence on U.S. federal aid

**Table 4:** Comparison of healthcare models and affiliated demographic trends between the Philippines and Puerto Rico. Source: Authors’ elaboration via online available data and governments’ websites.

Factor	The Philippines	Puerto Rico
**Healthcare Model**	Universal Health Care (UHC) system with mixed public–private involvement	US-based Medicaid and Medicare; limited access for undocumented residents
**Aging Population Policies**	Early-stage eldercare planning; continued reliance on family-based care	Advanced aging society with inadequate eldercare infrastructure; reliance on family caregiving
**Workforce Migration**	Mass healthcare worker migration abroad through Bilateral Labor Agreements and direct recruitment by foreign nations	Mass migration to the U.S. mainland, driving severe healthcare worker shortages
**Economic Sustainability of Healthcare System**	Fragmented financing after UHC implementation; weak rural infrastructure and low retention of physicians in deployment programs	Financial instability due to economic decline and reliance on U.S. federal aid; geographic barriers and emigration further reduce rural healthcare access

**Table 5:** Comparison of healthcare models and affiliated demographic trends between South Korea and Puerto Rico. Source: Authors’ elaboration via online available data and governments’ websites.

Factor	South Korea	Puerto Rico
**Healthcare Model**	Universal healthcare (NHI) with LTCI for elderly care; integration of AI and telemedicine to expand services	US-based Medicaid and Medicare; limited access for undocumented residents; constrained technology due to budget limits
**Aging Population Policies**	Extensive elderly care programs, though fiscally strained	Limited eldercare services with reliance on family caregiving
**Workforce Migration**	Import of foreign caregivers through the Employment Permit System (EPS)	Mass emigration, leading to a severe healthcare worker shortage
**Economic Sustainability of Healthcare System**	Heavily subsidized system facing sustainability challenges from rising long-term care costs	Financial instability due to economic decline and reliance on U.S. federal aid

## Data Availability

No new data was created or analyzed in this study.
